# Philip Morris International’s Formula 1 Sponsorship-Linked Marketing: Transformation From Marlboro to Mission Winnow

**DOI:** 10.1093/ntr/ntad177

**Published:** 2023-09-13

**Authors:** Timothy Dewhirst, Wonkyong Beth Lee, Lauren Czaplicki

**Affiliations:** Department of Marketing and Consumer Studies, Gordon S. Lang School of Business and Economics, University of Guelph, Guelph, ON, Canada; DAN Department of Management and Organizational Studies, Western University, London, ON, Canada; Department of Health, Behavior and Society, Bloomberg School of Public Health, Johns Hopkins University, Baltimore, MD, USA

## Abstract

**Introduction:**

Transformation describes a dramatic modification in appearance or character. Philip Morris International (PMI)’s sponsorship-linked marketing of Formula 1 auto racing is illustrative of transformation. The company’s flagship cigarette brand, Marlboro has been replaced as the identified partner by their newly developed brand, Mission Winnow. This study examines the tobacco company’s marketing objectives for transforming the brand identity of its Formula 1 Ferrari race team partnership.

**Aims and Methods:**

We provide a case study, and our method of qualitative enquiry is textual analysis. We review marketing planning documents from Philip Morris, which would normally be proprietary, but are publicly accessible because of litigation. Additionally, we review Mission Winnow’s social media posts, over a 3-year span, from the brand’s Facebook, Instagram, and Twitter accounts.

**Results:**

PMI initiated its Formula 1 sponsorship in 1972. Through Marlboro, the company’s sponsorship-linked marketing was largely centered on building brand image and reinforcing Marlboro’s brand identity of rugged masculinity, independence, heroism, and adventure. When Mission Winnow replaced Marlboro as the identified brand sponsor in 2018, the company’s marketing communication shifted to highlighting transformation, progress, open dialogue, teamwork, innovation, technology, and science.

**Conclusions:**

Despite Article 5.3 of the World Health Organization’s Framework Convention on Tobacco Control (WHO FCTC) calling for Parties to protect public health policies from the commercial and vested interests of tobacco companies, PMI still seeks to be an important stakeholder in such consultations, including those pertaining to harm reduction. Mission Winnow’s sponsorship-linked marketing points to a larger company narrative about trying to gain or reclaim legitimacy and credibility.

**Implications:**

PMI’s continued sponsorship of Formula 1 is a strategic means of drawing attention to the company’s “next-generation products” and communicating their supposed “transformation.” The company’s sponsorship-linked marketing initiatives point to a need for Parties to enforce Article 13 of the WHO FCTC, which calls for a comprehensive ban on tobacco advertising, promotion, and sponsorship.

## Introduction

A tweet from Philip Morris International (PMI), dated July 14, 2022, indicates that “Nobody has ever tried to #Unsmoke The World. We’re going into completely unchartered territory. Our CEO shares a bold statement regarding our transformation.” PMI goes on to largely present their transformation as getting existing users of cigarettes and combustible tobacco to switch to noncombustible products that are positioned as harm-reduced. PMI has adopted harm reduction in their public relations initiatives and marketing communication, including their annual reports and presentations to investors where self-styled “next generation products” are showcased.^1,2^ Moreover, PMI established a Foundation for a Smoke-Free World in 2017, which was supported by funding of roughly $1 billion and a stated mandate “to end smoking in this generation.”^3^ Still, from a marketing standpoint, such publicly stated corporate ambitions are questionable, as companies such as PMI have fundamental goals of maximizing sales, profits, and returns to shareholders. In seeking their imperative business goals, harm reduction is unlikely to be realized as “next generation products” are marketed to attract new users, encourage dual use (eg, being used when cigarette smoking is inconvenient or not allowable), and generally discourage quit attempts.^4^ While critical attention has been given to tobacco industry transformation initiatives such as PMI’s Foundation for a Smoke-Free World,^5,6^ the company’s continued sponsorship of Formula 1, and more specifically, partnership with the Ferrari race team, has been largely overlooked as another strategic means of drawing attention to their “next generation products” and communicating their supposed “transformation.” In this paper, we provide a case study of PMI’s Formula 1 sponsorship-linked marketing and reveal how the company’s marketing communication efforts have evolved to convey “transformation.” Whereas PMI’s flagship cigarette brand, Marlboro served as the longstanding sponsor or identified partner, a newly created brand, Mission Winnow, became apparent in 2018. While the World Health Organization’s Framework Convention on Tobacco Control (WHO FCTC) calls for a comprehensive sponsorship ban, significant investments in Formula 1 persist among multinational tobacco companies such as PMI.

*Branding* describes a name, term, symbol, or design to identify and give differentiated meaning to a product, service, or organization. *Brand identity* is a related and important marketing concept, being defined as “a unique set of brand associations that the brand strategist aspires to create or maintain. These associations represent what the brand stands for.”^7^ With the changing brand representation from PMI as sponsor of the Ferrari Formula 1 race team, and *transformation* describing a dramatic modification in appearance or character, our case study provides useful insight. Typical marketing thought for successfully communicating brand identity is to have promotional messages that are continuous and consistent.^7–9^ Nevertheless, PMI’s legal and political environment has apparently been a catalyst in the company’s atypical marketing approach of transforming the brand identity of its Formula 1 Ferrari race team partnership. Here, we provide a case study that gives a historical account of PMI’s transformation from Marlboro to Mission Winnow wherein we examine the company’s marketing objectives for transforming the brand identity of its Formula 1 Ferrari team partnership. Case study research which focuses on a single example such as a particular corporation or industry can be very useful. For example, it can be used to examine extreme exemplars or where there are unusual opportunities for research access.^10^ PMI is the world’s largest private tobacco firm and producer of Marlboro, which is the best-selling cigarette brand globally, and represents an extreme exemplar.^11^ Moreover, among Formula 1 race teams, Ferrari has a longstanding partnership with PMI and is regarded as “*the* F1 brand among brands.”^12^ Our case study includes the analysis of internal marketing planning documents from PMI, which are normally proprietary but made public from litigation, and exemplifies atypical research access.

## Methods

Our method of qualitative enquiry is textual analysis wherein “texts” may include the study of documents and media content (eg, advertising, promotions, and publicity).^13^ For our purposes, “texts” are regarded as objects to be examined and deconstructed; and, in doing so, we consider the words used, as well as pictorial elements. We reviewed internal corporate documents from PMI and the online Truth Tobacco Documents Library (https://www.industrydocumentslibrary.ucsf.edu/tobacco/) served as our key source. Use of this database for research purposes is well established.^14–16^ The period of analysis is from the late 1960s to 2022. The starting point reflects when tobacco companies increasingly shifted their promotional spending to sponsorship-linked marketing once traditional cigarette advertising was no longer allowable in the broadcast media in several jurisdictions.^17^ We generated documents for review from the Philip Morris collection by using a variety of search terms (eg, “Marlboro AND Sponsorship AND Formula One,” “Marlboro AND Ferrari AND Racing,” and “Marlboro AND auto racing”). Search terms generated a universe of documents for consideration, which prompted an initial assessment to distinguish those documents pertinent and applicable to our research questions. Thereafter, documents were organized chronologically to create a timeline of events, and common themes and codes were identified based on our review. Moreover, we triangulated internal corporate documents from our case study with multiple sources of evidence,^18,19^ including the company’s investor reports and webcasts, circulated newsletters, brand websites, as well as trade press.

Further supplementing our review of corporate documents, we conducted an exploratory analysis of Mission Winnow’s brand image and sponsorship activities on social media. We reviewed publicly available posts from Mission Winnow’s official Facebook (September 2018–May 2021), Instagram (October 2018–May 2021), and Twitter (May 2018–May 2021) accounts that were linked to the brand on its official website (https://www.missionwinnow.com/en/). Mission Winnow also maintains a LinkedIn account, but it was excluded from our analysis because LinkedIn is a platform tailored towards career professionals and not used as widely by the general public as observed for Facebook, Instagram, and Twitter. The starting point of the analysis reflected each social media account’s first available post; the endpoint of the analysis reflected those postings available on the account until May 1, 2021. For the subsequent analysis, initially conducted in May 2021, we reviewed all primary Mission Winnow posts—including copy and visuals—on each platform and documented the presence of the following themes across the entire body of posts from each account: *Sponsorship, Technology, Masculinity, Co-branding, Leadership, Transformation, Innovation*, *Communication*, and *Teamwork*. These categories were informed by theory and industry document review (eg, *Sponsorship*, *Technology*, *Masculinity*, *Co-branding, Innovation*), but also emerged from a holistic review of social media content (eg, *Transformation, Communication, Teamwork*). We describe the content of posts that fall into these categories across Facebook, Instagram, and Twitter and provide exemplary posts for each category.

## Results

Through its Marlboro cigarette brand, PMI initiated its Formula 1 sponsorship in 1972.^20^ With Marlboro (cigarettes) initially being the identified brand, PMI’s sponsorship-linked marketing was largely centered on building brand image. Marketing planning documentation identifies a primary objective as “communicate and extend the contemporary imagery of Marlboro Racing to reinforce Marlboro’s brand characteristics of masculinity, independence, and adventure.”^21,22^ Ellen Merlo, as Vice-President of Marketing Services at Philip Morris, indicated, “We perceive Formula One and Indy car racing as adding, if you will, a modern-day dimension to the Marlboro Man. The image of Marlboro is very rugged, individualistic, and heroic. And so is this style of auto racing. From an image standpoint, the fit is good.”^23^ Strategically, Marlboro and motor racing are regarded as congruent symbolically; according to Philip Morris documentation, “overlaps core imagery of confidence, determination, masculinity, independence, and control.”^21^ For Marlboro and auto racing, “the traits associated with the drivers are consistent with those often mentioned for the cowboy—masculinity, confidence, strength, determination, independence, and skill.”^21^ Moreover, Formula 1 sponsorship facilitated Marlboro being associated with an international or global sport as well as excitement, action, risk, technology, and market leadership.^24,25^ In the Philippines, for example, Philip Morris documentation from the 1990s shows, “The American cowboy and his lifestyle continue to be the backbone of Marlboro advertising … However, motorsports have provided a viable alternative opportunity for strengthening appeal to the key young adult segment, whose predispositions are directly addressed by the excitement and action of motor racing.”^24^

PMI’s Formula 1 sponsorship and partnership with the Ferrari race team began in 1984. Previously, the company’s sponsorship commitments included Marlboro signage alongside the racetracks; at the race team level, Marlboro was first a sponsor of McLaren before becoming a partner of Ferrari.^20^ Early in their new partnership, Philip Morris pursued a possible licensing agreement for the launch of a Ferrari-branded cigarette, wherein corporate documentation identified the prospective target market as young adult males (18 to 34 years of age) who were seeking a brand with macho connotations and high-technology imagery.^26,27^ Instead, however, Philip Morris strategically pursued their Marlboro brand being linked with the positive brand associations of Ferrari, which also include sophistication, innovation, premium quality, and speed (power and excitement).^28,29^ Ferrari and Marlboro were seen as complementary brands aesthetically because they are both associated with horses and the color, red (the Formula 1 Ferrari race car is red).^30^ Ferrari was recognized as possessing high brand awareness, and associated with premium quality as well as being overtly masculine, innovative, and technological.^27,30^ The brand attributes of Ferrari aligned well with the desired brand associations of Marlboro.

**Figure 1.**
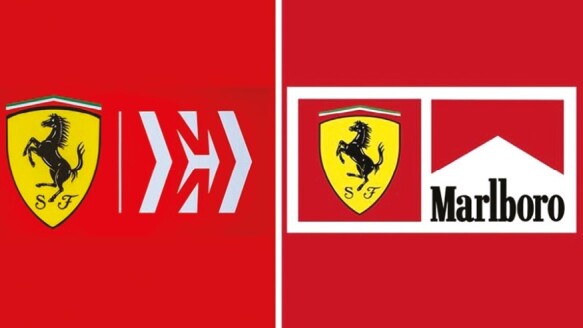
Philip Morris International demonstrated a transformation in brand identity with Mission Winnow replacing Marlboro as the identified sponsor or partner of the Ferrari Formula 1 race team.

In 2015, PMI extended its notable investment in Formula 1 sponsorship and the Scuderia Ferrari race team. Thereafter, “Mission Winnow” branding began appearing on Ferrari’s Formula 1 cars and drivers’ uniforms, which was first visible during the Japanese Grand Prix in October 2018. During the 2019 Formula 1 season, there was sporadic use of Mission Winnow branding during Formula 1 events, apparently in accordance with regulations regarding what representations were allowable based on where the race was situated. Whereas Mission Winnow branding was not permissible for races in Australia and Canada because of the enforcement of policy, such branding was highly visible for the races held in Brazil, China, Mexico, and Vietnam. A transformation in brand identity was obvious though with Mission Winnow replacing Marlboro as the identified sponsor as seen in Figure 1.

**Figure 2.**
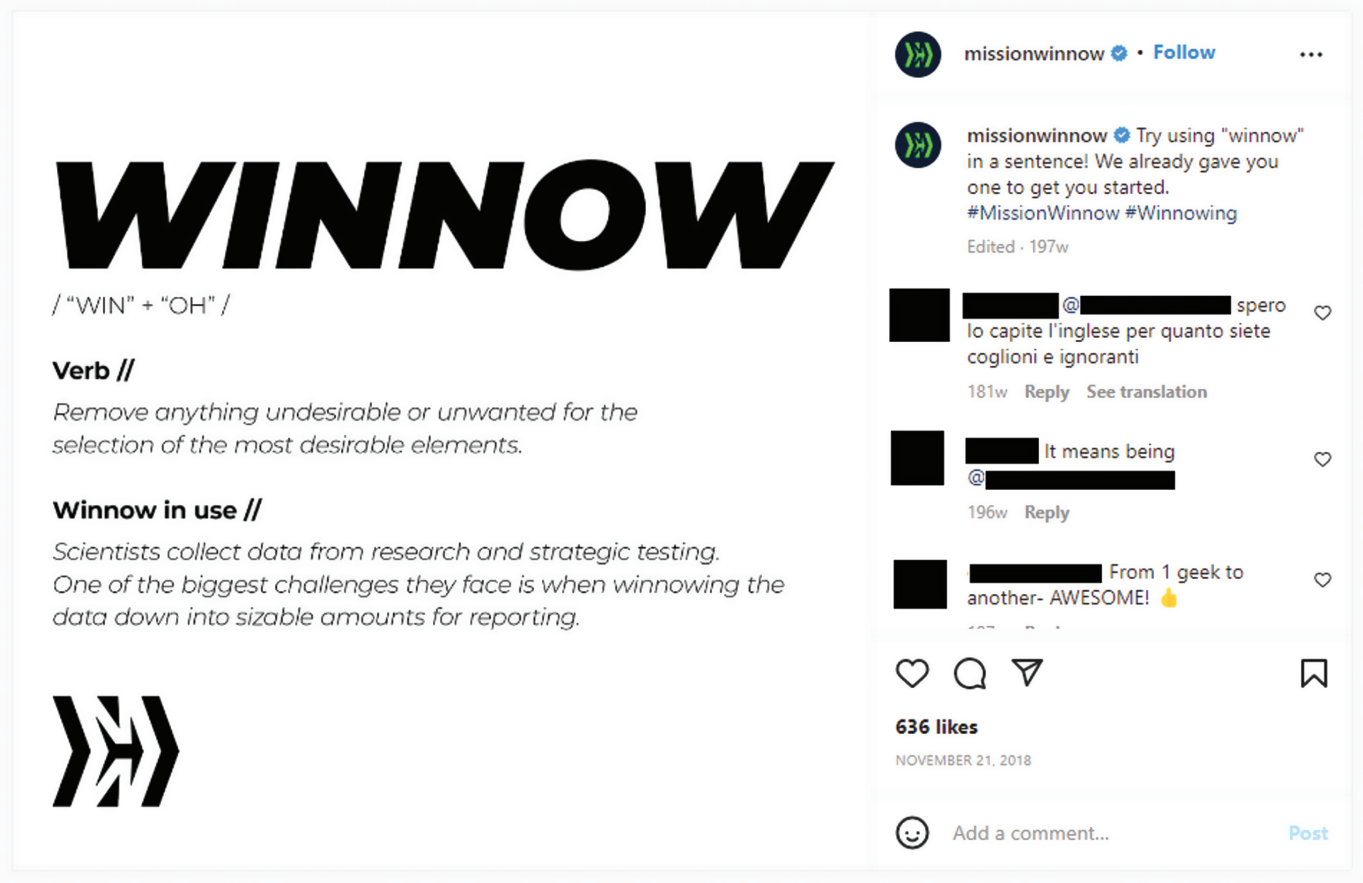
A social media post for Mission Winnow—dated November 21, 2018—presents “winnow” as a verb and defines the term.

Mission Winnow is registered as a trademark by PMI for “use with respect to tobacco products,”^31^ with a publicly stated aim of “developing and testing less harmful alternatives to smoking.”^32^ The brand name suggests taking on an important assignment as “mission” is synonymous with a task or undertaking. “Winnow,” meanwhile, is synonymous with examining, inspecting, or sorting through something. “Winnowing” describes sifting, separating, isolating, or filtering. Early social media posts for Mission Winnow often included the idea of “winnowing” away undesirable or unwanted elements for improved and efficient technology designs. One post—as seen in Figure 2—presented “winnow” as a verb (indicative of an action) and defined it as: “Remove anything undesirable or unwanted for the selection of the most desirable elements.” The post thereafter offered a sentence with “winnowing” in use (“Scientists collect data from research and strategic testing. One of the biggest challenges they face is when winnowing the data down into sizable amounts for reporting.”). Readers or followers were encouraged to also try using the word in a sentence. By providing an explicit definition that resembled what would be found in a dictionary, the pronunciation of the word was presented as “Win” and “Oh,” which is indicative of success and victory, as well as exhilaration and perhaps astonishment of what has been achieved. The Mission Winnow logo includes the letters “M” and “W,” yet appears to resemble an arrow that is directed at moving forward (ie, making progress).

When introduced in 2018, Mission Winnow branding appeared in black or white (against a red Ferrari car), yet its logo and representation changed to florescent bright green in 2021. The surprising branding change prompted many questions, which PMI spokesperson, Tommaso Di Giovanni justified as: “Much like a green screen does for film, the green arrow represents possibility and opens the opportunity to imagine new horizons.” Additionally, he emphasized that, “Mission Winnow focuses on promoting (and) driving positive change, fostering engagement, and opening conversations around the role that science, technology, and innovation can play in helping to address a range of societal challenges—now and into the future.”^33^ Curiously, the new bright green of Mission Winnow resembles Ferrari’s Verde Germoglio, which was introduced in the 1960s with the Ferrari Dino.^34^ While the color choice may be coincidental, it may also be indicative of PMI and Mission Winnow’s commitment as sponsor of Scuderia Ferrari, who are presented as “the most successful team in Formula 1 History, constantly winnowing and innovating” and being “a story of continual reinvention!”^35^

The Mission Winnow brand is marketed as being about transformation, progress, open dialogue, and innovation, which aligns PMI with technology and science. The Mission Winnow brand identity complements attributes associated with Formula 1. According to a Mission Winnow social media posting attributed to Jacek Olczak, PMI’s Chief Operating Officer, “For us at PMI, Mission Winnow is about how we are transforming as a company and what we believe in. It is a global communication platform encouraging people come together to drive & promote progress, and build constructive conversations around the topic of innovation. Nothing to do with brands or products. That is our promise.” This social media posting corresponded with Mission Winnow’s support and sponsorship of an event hosted by *The Economist* where Jacek Olczak was among the panelists. The event was entitled, “Defying conventional wisdom: Promoting a fact-based approach.”

Many social media posts for Mission Winnow link ideas of *technology* and *communication* to PMI’s efforts to transform and evolve as a company. One post, attributed to Miroslaw Zielinski, Mission Winnow’s President of Science and Innovation, states, “Mission Winnow is about driving change by constantly searching for better ways of doing things.” Another Mission Winnow post claims, “Going past our limits to positively impact the world beyond.” An Instagram post from Gizelle Baker, PMI’s Director of Scientific Engagement, reads: “Harm reduction is a business priority for PMI. We need to look at innovation to transform our entire company away from its past to become a technology company of the future.” Other posts discuss how open communication, in addition to direct dialogue with organizations, is fundamental to allowing Mission Winnow and PMI to achieve transformation. In one Facebook post, Mission Winnow’s Global Communications Director, Bryson Thornton, states that, “Mission Winnow is not just about PMI and our science. It’s also about how organizations and people can help drive us forward. When people come together and start talking, that’s when magic happens.” The ethos of these posts is carried over into Mission Winnow's social media content about the Formula 1 team, drawing parallels to how the racing team uses cutting-edge technology to be a leader in the field and how open communication and teamwork are upheld values of the racing team and drivers. The social media posting from Mission Winnow, featuring Bryson Thornton, also claims, “At PMI we want to be transparent, show our vision for the future to everyone that is willing to listen.”

Mission Winnow’s social media posts often contained a combination of the content categories, with *technology*, *transformation*, and *innovation* frequently linked. One exception was *masculinity*, where imagery of independence and rugged masculinity traditionally associated with Marlboro was noticeably missing from Mission Winnow’s posts. Rather, drivers were often portrayed in the context of being part of a team and having open and trusting relationships and communication with their teammates. *Sponsorship*-related content predominantly included posts that promoted racing events or looked in-depth at getting a car ready to race. A noticeable number of posts across platforms made links between PMI, Mission Winnow, and their sponsored teams when discussing advancements in industry and racing *technology*, *communication* and open-mindedness, and the importance of *teamwork*. Many Mission Winnow posts also linked *technology* and *communication* to PMI’s efforts of *transformation* and evolution as a company and their *innovation* efforts to create new technologies and reduce harm. According to a Mission Winnow post, “Everyone benefits from innovation. Our scientific solutions can be applied outside our field of excellence to positively impact other industries.” *Co-branding* was present in *sponsorship* imagery where cosponsors like Shell are visible in photos featured in social media posts. PMI’s partnership with Shell is longstanding. When Marlboro was still the identified sponsor of the Ferrari race team, both Marlboro and Shell livery appeared on the race car. Moreover, PMI and Shell had coadvertising agreements in place. In the Philippines during the early 1990s, for example, Shell “invited Marlboro to set up merchandising. They have shown and expressed preference for F1-related merchandising.”^24^ PMI’s partnership with Shell reflects both being sponsors of the Ferrari race team; still, both companies are also more likely to further leverage their partnership because they share common business interests with Shell overseeing many retail locations (eg, convenience stores), where PMI products are sold. Results from our social media review are summarized and described in Table 1, with exemplar images available at https://osf.io/78yqz/?view_only=462232ed9e8a49c9ab3695642974480c.

**Table 1.** Description of Main Categories Regarding Social Media Content Across Mission Winnow’s Facebook, Instagram, and Twitter Accounts Derived From an Exploratory Analysis of Posts Made Over a 3-Year Period (May 2018–May 2021)

Category definition	Content description
Sponsorship *Posts that highlight Mission Winnow’s relationship, support, and partnership with Scuderia Ferrari, and Ducati racing teams*	Posts that promote racing events and racing team activities for Scuderia Ferrari and Ducati-sponsored teams. Posts that describe the drivers as part of the Mission Winnow family. Event sweepstakes and giveaways of driver post-race photos.
Technology *Posts that highlight technological advances and/or how technology advances social progress*	Posts that celebrate a variety of technological advances across fields (eg, Braille reading system, artificial intelligence); early posts often included the idea of “winnowing” away undesirable or unwanted elements for improved and efficient technology designs Posts that document how Philip Morris International and Scuderia Ferrari are using science to advance technology in their field. Posts that discuss the specific role of technology in “harm reduction.”
Masculinity *Posts that feature Scuderia Ferrari and Ducati racing drivers*	Images of drivers exhibiting overt masculinity or rugged independence were not present in Mission Winnow content reviewed; in contrast, posts often show drivers working together as a team and show the emotional experience of racing.
Co-branding *Posts that contain cross-promotion of other brands, including celebrities*	Posts that promote Mission Winnow and Philip Morris International presence at events hosted by The Economist. Posts that promote the Mission Winnow interview series *The Dominant Ones* which feature celebrities like Dwayne Wade, Dr. Oz, etc.
Innovation *Posts that highlight new ideas or projects meant to innovate on an existing project or problem*	Posts that discuss Philip Morris International’s future plans and being responsive to change and advancing progress (eg, “The future is always being created, we can’t wait to show you what we’ve been working on!”). Posts the promote collaborative initiatives with different start-ups to help create Philip Morris International products.
Transformation *Posts that highlight changing or evolving over time*	Posts that discuss how Mission Winnow reflects an evolution and change in Philip Morris International (eg, “We can stay true to ourselves and evolve”). Posts that discuss change as positive and needed for growth, including embracing new ideas and new information/studies that can change opinion.
Communication *Posts that highlight the role of discussion, debate, and general need for communication*	Posts that highlight the importance of listening, understanding, and being open-minded, including video clips of Scuderia Ferrari or Ducati drivers talking about communication. Posts that discuss civility in debate and how multiple points of view should be considered, often include quotes or videos of Philip Morris International executives.
Teamwork *Posts that highlight how a team works together*	Posts that celebrate the value of experience and talent on teams, including Philip Morris International, Mission Winnow, and Scuderia Ferrari teams.

## Discussion

As a sponsor of Formula 1, PMI could further build Marlboro’s brand image by being associated with the masculine, independent, adventurous, and heroic attributes of the sports property. Certain symbolic links get highlighted with the strategic aim that the sports property’s image associations are credibly transferable towards the sponsoring brand.^36,37^ Marlboro is commonly promoted as an expression of rugged masculinity, independence, and power or leadership.^38,39^

Still, PMI faced increasingly stringent regulatory environments, which extended to their permitted use of sponsorship-linked marketing. For example, legislation was implemented in the United Kingdom, where most Formula 1 teams are based, that effectively banned tobacco advertising at sports events in 2005.^40^ Additionally, the WHO FCTC took effect in 2005. Article 13 of this global public health treaty, which now includes 182 Parties and covers more than 90% of the world’s population, calls for a comprehensive ban on tobacco advertising, promotion, and sponsorship.^41^ Facing such a reality and public pressure, Formula 1’s governing body, Fédération Internationale de l’Automobile, prohibited tobacco sponsorship from the end of 2006.^40^ PMI’s Formula 1 sponsorship investments persisted, however, despite such policies. From 2007 to 2010, the Ferrari race car featured barcode designs that exemplified alibi Marlboro branding.^42^*Alibi marketing* refers to strategic attempts at extracting a brand identity from its key components, such as using the red and white colors associated with the Marlboro brand as a substitute for the brand’s conventional logo or trademark.

Ultimately, however, PMI changed its brand identity from Marlboro to Mission Winnow—undergoing a “transformation”—for its sponsorship-linked marketing activities. Accordingly, our results reveal that different image dimensions or attributes of Formula 1 became highlighted. One pronounced difference is that “independence” was an underscored theme when Marlboro was the sponsoring brand, yet “teamwork” became emphasized once Mission Winnow was the identified sponsor. While Marlboro sponsorship-linked marketing was more likely to celebrate the individual driver (eg, in action in their race car or seen on the podium demonstrating their success or heroism), marketing communication for Mission Winnow tends to feature the pit crew and indicate the importance of collaboration and working with others towards a common goal. This change of emphasis reflects being true to what the Marlboro and Mission Winnow brands are meant to represent, but also points to a larger PMI narrative about trying to gain or reclaim legitimacy.

Mission Winnow's marketing communication aims to improve PMI’s credibility by being seen as trustworthy, showcasing their partnerships, engaging in science and open dialogue, and presenting “facts.” Companies such as PMI seek to present “science” to inform public health policies, including those relating to harm reduction, yet it can be unclear if the conclusions are based on independent research. Several scholars championing the adoption of harm reduction have undisclosed interests, which has been identified as a longstanding ethical issue.^43,44^ A research article was recently retracted from the *European Respiratory Journal*, for example, at the request of the editors and publisher once learning that the authors had undisclosed interests, including one author’s funding from the PMI-supported, Foundation for a Smoke-Free World.^45^ Marketing scholars have generally cautioned that, “Business organisations with proprietary aims and profit-motivated goals own and control most of the data, most of the platforms, and most of the data analysis tools in the world—a situation that does not bode well for the future of open research.”^46^

Another pronounced difference observed between Marlboro and Mission Winnow marketing communication pertains to how “masculinity” is expressed. It is important to recognize that PMI’s association or link with the Formula 1 sports property now spans 50 years, while the concept of masculinity and norms about its expression constantly evolves (ie, the concept is not regarded as static). Masculinity represents what is regarded as appropriate for men (with respect to roles, behavior, and attributes) in each society and varies by culture and over time.^47^ General expectations of manliness will be different in the 1970s compared to today. Historically, Formula 1 is recognized as an overwhelmingly masculine sport. Culturally, Formula 1 has previously featured men holding to a patriarchal version of masculinity, wherein the sports property was dubbed a “Playboy’s Playground.”^48^ Legendary driver, James Hunt, who was sponsored by Marlboro, was well known for his prominent skill on the track as well as a notorious lifestyle as Formula 1’s ultimate playboy. Additionally, Bernie Ecclestone, the former longstanding chief executive of Formula 1, made controversial comments like “women should be dressed in white like all the other domestic appliances.”^49^ However, recent dialogue about gender identity, diversity, and equity has the “Marlboro Man” being questioned as the ultimate model of masculinity. Brands targeting men such as Gillette recognize that the traditional rugged masculinity commonly associated with the Marlboro Man can be problematic and are engaged in a fast-evolving cultural discourse about what it means to be a man in contemporary society.^50^ Men historically dominated as the actors of Formula 1, but recent efforts to attract more diverse fan bases are apparent. In 2018, Liberty Media, as new controlling owners of Formula 1, ended the practice of using “grid girls.” Also, Formula 1’s governing body, Fédération Internationale de l’Automobile (FIA), initiated the FIA Girls on Track—Rising Star program, which aims to identify and develop the best 12- to 16-year-old female auto racing drivers and partners with companies that include the Ferrari Driver Academy.^51^

As aforementioned, the recent portrayal of communication and teamwork in Mission Winnow’s social media posts about Formula 1 drivers contrasts with the historic portrayal of independence and rugged masculinity that was often associated with Marlboro. This could signal PMI’s further transformation away from the brand’s iconic imagery of the Marlboro Man, which represents an independent cowboy, to a more contemporary expression of masculinity where emotions, being “inspired” by others, and teamwork are allowed in the service of innovation. Still, the technology sector is dominated by men too, with Silicon Valley still regarded as a “boys’ club” whose executives are among the richest and most powerful people in history. The CEOs of Amazon, Apple, Google, and Microsoft, for example, have continuously been men.^52^ Innovation and technology are now central attributes of Formula 1 that PMI looks to highlight and further link with their Mission Winnow brand as sponsor; accordingly, the brand remains associated with masculinity, but with a more contemporary outlook or representation.

### Limitations and Future Research

Given that this study exemplifies interpretive research, we acknowledge that different analysts will not necessarily generate results that are replicable and reproducible.^53^ As textual analysis includes the construction of meaning, it is a subjective process and analysts may not always agree on their findings. The skills and experience of the analysts provide a possible safeguard against criticisms concerning the validity of such analyses.^15,54,55^ Our research team has considerable experience with textual analysis specific to the tobacco industry. Additionally, multiple investigators in a research team are more likely to generate novel insights, while a triangulation role is served when there is a convergence of observations, thus building confidence in the findings.^18^

A second limitation pertains to the review of corporate documents, made public from litigation, and our periodization scheme. Despite also seeking contemporary marketing planning documents from the online Truth Tobacco Documents Library, search terms typically generated considerable documents for review from the 1970s, 1980s, and 1990s. These marketing planning documents provided important insight regarding Marlboro’s sponsorship of Formula 1 but did little to inform about Mission Winnow becoming an identified sponsor recently. Consequently, we examined Mission Winnow's social media posts to supplement our review of corporate documents. Posts were reviewed on the platforms and not downloaded. We did not take inventory of individual posts; therefore, we are unable to provide an accounting for the total number of posts. Still, most of the platforms posted daily or near daily.

The scope of this research pertained to PMI strategic aspects wherein we provide a “marketing management” perspective. The purpose of our research did not include examining the effects on consumers or the recipients of marketing communication. Future research that analyzes consumer and other stakeholder perspectives, including their engagement with social media posts, would be fruitful. Moreover, using machine learning classifiers in future research can offer deeper insight into Mission Winnow’s social media presence, including temporal trends in post content.

We have presented a case study focused on a single example: PMI and the company’s sponsorship-linked marketing initiatives pertaining to Formula 1. Although the PMI tweet which we presented at the opening of this paper suggests that the company’s proclaimed transformation is a unique one, direct competitor, British American Tobacco (BAT), also continues to sponsor Formula 1. Like PMI’s “Mission Winnow,” BAT has created a new brand, “A Better Tomorrow,” for sponsoring the McLaren team that facilitates the promotion of the company’s “next generation products.” According to BAT’s website, “BAT and McLaren share a passion for technology, innovation and design.”^56^ Future research that presents case studies of PMI’s key competitors and their comparative marketing practices would be informative.

### Implications

Through sponsorship, the Ferrari and McLaren race teams received an estimated $95 million from tobacco companies during the 2019 Formula 1 season,^40^ despite a tobacco sponsorship ban supposedly being in effect. PMI’s Chief Operating Officer, Jacek Olczak’s claim that Mission Winnow has “nothing to do with brands or products” is highly questionable. The tobacco industry has a longstanding history of circumventing policy through sponsorship-linked marketing, including the use of alibi marketing or maintaining tobacco brand visibility through cosponsors’ advertising initiatives.^17,42^ PMI’s transformation from Marlboro to Mission Winnow serves as another example of evading policy stipulations.

The WHO FCTC, which is legally binding for those countries that ratify the treaty, “requires parties to adopt a comprehensive range of measures designed to reduce the devastating health and economic impacts of tobacco” (see http://www.fctc.org for details). To date, 182 Parties have signed and ratified the WHO FCTC. Article 5.3 of the WHO FCTC mandates that Parties protect public health policies from the commercial and vested interests of tobacco companies. Article 13 calls for a comprehensive ban on tobacco advertising, promotion, and sponsorship; guidelines for the implementation of Article 13 also identify that corporate social responsibility activities are forms of tobacco advertising and promotion covered by the WHO FCTC’s stipulations.^57^ These stipulations are meant to be applicable to the tobacco industry’s noncombustible and “next-generation products.” The recommendation of a WHO report from the sixth session of the Conference of the Parties is “an outright ban on ENDS (electronic nicotine delivery systems) advertising, promotion, and sponsorship is preferable to the implementation of voluntary codes on ENDS marketing, given the overwhelming evidence that similar codes for tobacco and alcohol products have failed to protect young people from such advertising.”^58^ Evidently, there is a need for more Formula 1 host countries, beyond Australia and Canada,^59^ to strictly enforce tobacco control policies, especially among those that are Parties of the WHO FCTC.

## Conclusions

Major multinational tobacco companies are investing substantial money into the sponsorship-linked marketing of Formula 1 auto racing as a means of conveying their supposed “transformation.” Historically, PMI’s sponsorship-linked marketing of Formula 1 was largely centered on building brand image and reinforcing Marlboro’s brand identity of rugged masculinity, independence, heroism, and adventure. When Mission Winnow replaced Marlboro as the identified brand sponsor in 2018, the company’s marketing communication shifted to highlighting transformation, progress, open dialogue, teamwork, innovation, technology, and science. Despite the WHO FCTC calling for Parties to protect public health policies from the commercial and vested interests of tobacco companies, PMI still seeks to be an important stakeholder in such consultations, including those pertaining to harm reduction. Mission Winnow’s sponsorship-linked marketing points to a larger company narrative about trying to gain or reclaim legitimacy and credibility.

## Data Availability

Data derived from a source in the public domain. The data underlying this article are available in Truth Tobacco Industry Documents, hosted by the University of California San Francisco Library, at https://www.industrydocuments.ucsf.edu/tobacco/; as well as posts from Mission Winnow’s Facebook, Instagram, and Twitter accounts, which are publicly available and in the public domain.

## References

[1] Edwards R , Hoek J, Karreman N, Gilmore A. Evaluating tobacco industry “transformation”: a proposed rubric and analysis. Tob Control. 2022;31(2):313–321. 35241605 10.1136/tobaccocontrol-2021-056687

[2] Peeters S , Gilmore AB. Understanding the emergence of the tobacco industry’s use of the term tobacco harm reduction in order to inform public health policy. Tob Control. 2015;24(2):182–189. 24457543 10.1136/tobaccocontrol-2013-051502 PMC4345518

[3] Foundation for a Smoke-Free World. Our Vision: Foundation for a Smoke-Free World, 2022. https://www.smokefreeworld.org/our-vision Accessed August 10, 2022.

[4] Dewhirst T. Co-optation of harm reduction by Big Tobacco. Tob Control. 2021;30(November):e1–e3. 32796080 10.1136/tobaccocontrol-2020-056059

[5] van der Eijk Y , Bero LA, Malone RE. Philip Morris International-funded “Foundation for a Smoke-Free World”: analysing its claims of independence. Tob Control. 2019;28(6):712–718. 30242044 10.1136/tobaccocontrol-2018-054278

[6] Legg T , Legendre M, Gilmore AB. Paying lip service to publication ethics: scientific publishing practices and the Foundation for a Smoke-Free World. Tob Control. 2021;30(e1):e65–e72. 33911028 10.1136/tobaccocontrol-2020-056003 PMC8606453

[7] Aaker DA. Building Strong Brands. New York: The Free Press, 1996: 68.

[8] Ries A , Ries L. The 22 Immutable Laws of Branding. New York: HarperCollins Publishers, 1998.

[9] D’Alessandro DF. Brand Warfare: 10 Rules for Building the Killer Brand. New York: McGraw-Hill, 2001.

[10] Eisenhardt KM , Graebner ME. Theory building from cases: opportunities and challenges. Acad Manage J. 2007;50(1):25–32.

[11] Philip Morris International. Building leading brands. 2023. https://www.pmi.com/investor-relations/overview/building-leading-brands. Accessed March 11, 2023.

[12] Fleming D , Sturm D. Media, Masculinities and the Machine: F1, Transformers and Fantasizing Technology at its Limits. New York: Continuum, 2011: 170.

[13] Travers M. Qualitative Research Through Case Studies. London: Sage Publications, 2001.

[14] Carter SM. Tobacco document research reporting. Tob Control. 2005;14(6):368–376. 16319359 10.1136/tc.2004.010132 PMC1748115

[15] Anderson SJ , Dewhirst T, Ling PM. Every document and picture tells a story: using internal corporate document reviews, semiotics, and content analysis to assess tobacco advertising. Tob Control. 2006;15(3):254–261. 16728758 10.1136/tc.2005.013854 PMC2564670

[16] Anderson SJ , McCandless PM, Klausner K, Taketa R, Yerger VB. Tobacco documents research methodology. Tob Control. 2011;20(suppl 2):ii8–i11. 21504933 10.1136/tc.2010.041921 PMC3085001

[17] Dewhirst T , Hunter A. Tobacco sponsorship of Formula One and CART auto racing: tobacco brand exposure and enhanced symbolic imagery through co-sponsors’ third party advertising. Tob Control. 2002;11(2):146–150. 12035009 10.1136/tc.11.2.146 PMC1763846

[18] Eisenhardt KM. Building theory from case study research. Acad Manage Rev. 1989;14(4):532–550.

[19] Yin RK. Case Study Research: Design and Methods, 3rd ed. Thousand Oaks, CA: Sage Publications, 2003.

[20] Philip Morris. Marlboro and motor racing. 1983: Bates no. 2501060185-2501060186.

[21] Philip Morris. Marlboro racing. 1998: Bates no. 2070683673-2070683709.

[22] Philip Morris. Marlboro Team Penske Racing. 1998: Bates no. 2071614261.

[23] The business of racing, Marlboro advertisement. The New York Times Magazine 1989;(July 9).

[24] Philip Morris Asia Incorporated. Formula One evaluation: Philippines. 1993: Bates no. 2504052837-2504052839.

[25] Analisi E Strategie Di Mercato S.r.l. (ASM). Ferrari image study: JOB N. 4654/LT management summary. Prepared for Philip Morris Europe. 1993(February):Bates no. 2501057286-2501057323.

[26] Dangoor DER. Project Red Ferrari trademark for the U.S. Interoffice correspondence of Philip Morris USA. 1987: Bates no. 2044201818.

[27] Tso D. Ferrari project. Interoffice correspondence of Philip Morris USA. 1987: Bates no. 2041510534-2041510544.

[28] Philip Morris. Proposed comments on Italy objectives 1991. 1991: Bates no. 2501056403-2501056409.

[29] Philip Morris. Adaptation marketing plan [Lausanne, Post-Vizzini]. 1992: Bates no. 2501060174-2501060183.

[30] Tso D. Ferrari – preliminary development strategy. Philip Morris, U.S.A. interoffice correspondence. 1986: Bates no. 2044201833-2044201835.

[31] World Health Organization. WHO urges governments to enforce bans on tobacco advertising, promotion and sponsorship, including in motor sport. 2019. https://www.who.int/news/item/14-03-2019-who-urges-governments-to-enforce-bans-on-tobacco-advertising-promotion-and-sponsorship-including-in-motor-sport. Accessed June 10, 2021.

[32] Mission Winnow. Breaking new scientific ground. 2021; https://www.missionwinnow.com/en/pmi/science-and-innovation-at-pmi/. Accessed May 10, 2021.

[33] Collantine K. Ferrari sponsor explains surprise change to bright green logo. RaceFans. 2021(March 11). https://www.racefans.net/2021/03/11/ferrari-sponsor-explains-surprise-change-to-bright-green-logo-01/. Accessed August 31, 2022.

[34] Lammers M. Complete rundown of green Ferrari shades. ROSSOautomobili. 2022. https://rossoautomobili.com/blogs/magazine/complete-rundown-of-green-ferrari-shades. Accessed May 27, 2022.

[35] Mission Winnow. Scuderia Ferrari. 2022; https://www.missionwinnow.com/en/scuderia-ferrari/. Accessed August 31, 2022.

[36] Gwinner KP , Eaton J. Building brand image through event sponsorship: the role of image transfer. J Adv. 1999;28(4):47–57.

[37] Prendergast GP , Poon D, West DC. Match game: linking sponsorship congruence with communication outcomes. J Adv Res. 2010;50(June):214–226.

[38] Aaker JL. Dimensions of brand personality. J Marketing Res. 1997;34(August):347–356.

[39] Hafez N , Ling PM. How Philip Morris built Marlboro into a global brand for young adults: implications for international tobacco control. Tob Control. 2005;14(4):262–271. 16046690 10.1136/tc.2005.011189 PMC1748078

[40] Vital Strategies. Driving Addiction: F1 and Tobacco Advertising. New York: Money Sport Media Limited; 2020.

[41] World Health Organization. WHO Framework Convention on Tobacco Control: Parties 2022; https://fctc.who.int/who-fctc/overview/parties. Accessed September 12, 2022.

[42] Grant-Braham B , Britton J. Motor racing, tobacco company sponsorship, barcodes and alibi marketing. Tob Control. 2012;21(6):529–535. 21821820 10.1136/tc.2011.043448 PMC3595501

[43] Soley LC. Smoke-filled rooms and research: a response to Jean J. Boddewyn’s commentary. J Adv. 1993;22(4):108–109.

[44] Soley LC , Feldner SB. Transparency in communication: an examination of communication journals’ conflicts-of-interest policies. J Commun Inq. 2006;30(3):209–228.

[45] Giannouchos TV , Sussman RA, Mier JM, Poulas K, Farsalinos K. Retraction notice for: “Characteristics and risk factors for COVID-19 diagnosis and adverse outcomes in Mexico: an analysis of 89,756 laboratory-confirmed COVID-19 cases.” Eur Respir J. 2020;57(3):2002144. in press. 10.1183/13993003.02144-2020 PMC7397951 32732325

[46] Kozinets RV , Scaraboto D, Parmentier M-A. Evolving netnography: how brand auto-netnography, a netnographic sensibility, and more-than-human netnography can transform your research. J Market Manage. 2018;34(3-4):231–242.

[47] Kimmel MS. Masculinity as homophobia: fear, shame and silence in the construction of gender identity. In: Gergen MM, Davis SN (eds.), Toward a New Psychology of Gender. New York: Routledge, 1997: 223–242.

[48] Pelletier A. The Grand Prix is a paradise for male entitlement. Feminist Current. 2015. https://www.feministcurrent.com/2015/06/10/the-grand-prix-is-a-paradise-for-male-entitlement/. Accessed August 29, 2022.

[49] ESPN. Ecclestone repeats “domestic appliance” quip. 2005. https://www.espn.com/racing/news/story?series=irl&id=2092194. Accessed August 29, 2022.

[50] Adams P. Goodbye, Marlboro Man: how marketers are breaking with traditional notions of masculinity. Marketing Dive. 2018. (August 20); https://www.marketingdive.com/news/goodbye-marlboro-man-how-marketers-are-breaking-with-traditional-notions/530094/. Accessed August 31, 2022.

[51] FIA Formula E. FIA girls on track. 2022; https://www.fiaformulae.com/en/championship/fia-girls-on-track. Accessed August 31, 2022.

[52] O’Mara M. Why can’t tech fix its gender problem? MIT Technology Review. 2022. https://www.technologyreview.com/2022/08/11/1056917/tech-fix-gender-problem/. Accessed August 30, 2022.

[53] Morse JM. “Perfectly healthy, but dead”: the myth of inter-rater reliability. Qual Health Res. 1997;7(4):445–447.

[54] Leiss W , Kline S, Jhally S. Social Communication in Advertising, 2nd ed. New York: Routledge, 1997.

[55] Penn G. Semiotic analysis of still images. In: Bauer MW, Gaskell G (eds.), Qualitative Researching with Text, Image and Sound. Thousand Oaks, CA: Sage Publications, 2000: 227–245.

[56] British American Tobacco. Our global partnership with McLaren. 2021; https://www.bat.com/abettertomorrow. Accessed May 10, 2021.

[57] World Health Organization. Elaboration of guidelines for implementation of Article 13 of the Convention. WHO Framework Convention on Tobacco Control, Conference of the Parties to the WHO Framework Convention on Tobacco Control, Third Session, Durban, South Africa, 17-22 November 2008, 2008. pp. 5–6.

[58] World Health Organization. Electronic nicotine delivery systems. Conference of the Parties to the WHO Framework Convention on Tobacco Control, Sixth Session, Moscow, Russian Federation, 13-18 October 2014. 2014. Document FCTC/COP/5/13, p. 11.

[59] Dewhirst T. The geopolitics of money versus morals: location, location, location of the Formula 1 race calendar. In: Chadwick S, Widdop P, Goldman MM (eds.), The Geopolitical Economy of Sport: Power, Politics, Money, and the State. London: Routledge, 2023: 235–241.

